# High snakebite underreporting rate in the Centre Region of Cameroon: an observational study

**DOI:** 10.1186/s12889-019-7363-3

**Published:** 2019-08-03

**Authors:** Désiré Tchoffo, Joseph Kamgno, Sévilor Kekeunou, Callixte Yadufashije, Hugues C. Nana Djeunga, Armand Séraphin Nkwescheu

**Affiliations:** 1 Faculty of Health and Life Science, Distant Production House University (DPHU), Delaware, USA; 2 Centre for Research on Filariasis and other Tropical Diseases (CRFilMT), Yaoundé, Cameroon; 3 Department of Public Health, Faculty of Medicine and Biomedical Sciences (FMBS), University of Yaoundé I, Yaoundé, Cameroon; 4 Parasitology and Ecology Laboratory, Department of Animal Biology and Physiology, Faculty of Science, University of Yaoundé I, Yaoundé, Cameroon; 5 INES-Ruhengeri-Institute of Applied Sciences, Muzanze, Rwanda; 6 Laboratory of Public Health for Biotechnology and Research, University of Yaoundé I, Yaoundé, Cameroon

**Keywords:** Epidemiological profile, Snakebites, Underreporting, Centre region, Cameroon

## Abstract

**Background:**

In Cameroon, since the first epidemiological week held in 2015, snakebites have been registered among Potential Epidemic Diseases (PED). In the Centre Region, the most densely populated of the country, weekly reports of snakebites are generated at health districts level for monthly data updates.

**Methods:**

To contribute to the better management of snakebite cases, an observational study was conducted to assess the snakebite reporting rate in the Centre Region of Cameroon. The results of this retro-prospective survey were confronted to those of the weekly epidemiological surveillance system, recorded in the PED regional data base.

**Results:**

The incidence of bites was relatively high (36.6 bites per 100,000 inhabitants), as well as the general attack rate (about 49 envenomations per 100 victims). The lethality recorded was 2.5% and the mortality was about 1 death per 100,000 inhabitants a year. The sex ratio was largely female biased (61.6%). The bites occurred mostly during the rainy season (73.0%). Bitten victims were mainly farmers (47.4%), and agriculture was the main risk factor. The comparative analysis of the data suggested a high non-reporting rate of snakebite cases (67.8%).

**Conclusion:**

Snakebite is an endemic condition in the Centre Region of Cameroon. Because of the high rate of non-reporting of cases, the collection of information from the registers of the health facilities only appears not enough to assess the real importance of envenomation in this Region.

## Background

After its integration into the large group of envenomations, snakebites have been included since June 2017 in the Neglected Tropical Diseases (NTDs) portfolio of the World Health Organization (WHO) [1]. Snakebite envenoming can cause paralysis leading to breathing and bleeding disorders, fatal hemorrhage, irreversible kidney failure and tissue damage that can cause permanent disability and limb amputation. The highest burden of this neglected condition occurs in Africa, Asia and Latin America, especially in countries where health systems are weak and medical resources sparse [2]. The clinical complications of snake bites can be very serious or even fatal. A cobra or Mamba bite can lead to death by asphyxiation due to respiratory paralysis within 6 h following the accident [3]. The great variability in the composition of snake venoms is responsible for the diversity of symptoms observed in the snakebite. This includes neurotoxicity, such as paralysis, skeletal muscle destruction, hemostasis disorders such as coagulopathies, edema, bleeding and necrosis [4]. During the rainy season in some African regions, snakes enter homes to search for shelters to be protected from precipitation, causing bites on their path, thus leading to the increase in hospitalization rates for snakebites above 10% [5]. With regard to the variety of biotopes and the environmental characteristics favorable to the development of snakes, Cameroon is the Central African country where most of the Herpetology studies had been conducted [6]. About 270 species of reptiles are known in Cameroon, of which 150 species of snakes, 32 representing a danger to humans [6]. Cameroon is among the five African countries teeming with so-called “snake pockets”, characterized by snakebite incidence rates greater than 400 cases/100,000 inhabitants/year [3]. Indeed, these figures are largely below the estimates of the International Society of Toxinology (IST), ranged between 2255 and 6206 annual cases of bites with a lethal charge around 33.8 to 265.9 cases in Cameroon [7]. This can be due to the difficulty in accessing health units in certain areas and the frequent use of other therapeutic routes. Also, the incidence of snakebites, morbidity and mortality associated, as well as the therapeutic routes followed by the victims remain poorly documented. Indeed, in 2011 for example, only 10% of the victims of snakebites had been able to benefit from the recommended health care due to non or poorly sensitized medical staff and the lack of anti-venoms in Cameroon [5].

During the year 2015, there was a quasi-absence of Anti-Venom Serum (AVS) in the purchasing centers, dispensaries and pharmacies of health facilities in Cameroon. In 2016, the distribution and consumption of the two anti-venom sera marketed following the national supply system recommendations for essential medicines remained low, despite the subsidy of 94% granted by the Ministry of Public Health to promote the accessibility to these sera by the victims of envenomation. The need to identify measures that can facilitate targeted actions for the distribution of AVS and the access of snakebite victims to quality care remains topical.

The objective of this study was to assess the real snakebites reporting rate in the Centre Region of Cameroon by comparing data observed in the field to those reported by the regional surveillance system and drawing the epidemiological profile of snakebites victims in the Region.

## Methods

### Study area

This study was conducted in the Centre Region of Cameroon, which covers an area of 69,005 km^2^ with an average density of population of 51.1 inhabitants/Km^2^ [4]. The Centre Region is one of the 10 administrative regions of the country. This Region is organized into 30 Health Districts (HD) (each HD shelters a District Hospital (DH)), 248 health areas (HA) and about 838 health facilities [8] where the victims of envenomation are referred to for better management, from the surrounding communities.

The environment of the Centre Region is of forest-savanna transition type, offering a suitable biotope conducive to the development of various species of snakes. This region is characterized by a Guinean-type equatorial climate with four-seasons, including two rainy seasons (mid-March to June and September to mid-November) and two dry seasons (mid-November to mid-March and July to August).

The Centre Region shelters the species of the two larger groups of snakes existing in Cameroon, the “Scolecophidiens” and the “Alethinophidiens” [6]. The absence of a real land policy, thus leading to the non-observance of the urbanization standards, the precariousness of most of the houses built with temporary materials, the important hydrographic network (including the Sanaga rivers and its tributaries), and the vast forested environment (which is the extension of the Congo Forest Basin) are supportive of the important diversity and abundance of snake species encountered in this region.

### Study population

The population of the Centre Region consists essentially in the Fang Akilan made up of several indigenous ethnic groups (Ewondos, Benés, Boulous, Fangs, Ntoumous, Mvaés, Etons, Manguissas, Ossanagas, Yezums, Mbidas, Mbanis, Yebekolos, Baboutés, Bafias, the Yambassas, Sos, Mveles, Banens, Tikars). In addition of these native ethnic groups, the Bassas, Bamilékés as well as the immigrants from other countries (Mali, Senegal, Nigeria, and China) constitute another important part of the population of the Centre Region. The main activity of these populations is subsistence agriculture, though most of the immigrants are traders.

Eligible individuals for this study were the victims of snakebites. Snake-bitten patients during the period of July 2015 to June 2016 in the Centre Region were enrolled in this study. Resident or migrating individuals within the selected health areas who were victims of a snakebite during the study period were included in the study. Any victim of snake bite during the study period and within the selected health area, but absent or inaccessible in the health areas during the data collection period or any victims who freely refuses to participate were not included in the study.

### Study design

Two sampling techniques were used in the framework of this study, (i) cluster sampling and (ii) snowball sampling.

Cluster sampling was based on the organization of the pyramid structure of the Cameroon health system. Indeed, the 30 health districts in the Region were visited, 10% of the health areas of each health district were randomly chosen, and 10% of the health facilities of each health area were visited at random. Snakebite victims who attended the selected health facilities and the health professionals working in these services were administered a questionnaire.

In each cluster (health area), the snowball approach was further used for identification of snakebite victims who did not attended any health facility, the sampling unit being households. The first line informants of this approach were traditional healers; the snakebites victims identified using this sampling technique were used for the identification of other victims. In addition, some snakebite patients in the hospitals also help in the identification of victims in the communities. The snakebites victims enrolled in this study were not only those injured by snake teeth or hooks, but also included the victims of the projection of the venomous substance in the mucous membranes or on the skin, as it is the case of some snakes like the spitting cobra attacking or defending themselves by projecting their venom into the eyes of their victims [6].

The data collection was carried out in two steps; a retrospective survey carried out in the targeted/selected health facilities, and a prospective survey carried out in health facilities and communities sharing the same geographical settings in the visited health areas.

### Retrospective survey

Consultation registers were screened, and snake bitten patient records from July 2015 to December 2015 (6 months) retrieved. The data of snakebites recorded in the regional synthesis of Potential Epidemic Disease (PED) during the same period were also retrieved.

### Prospective survey

Snake-bitten victims were recruited from January 2016 to June 2016 in the health facilities as they were bitten. A questionnaire aiming at identifying the enrollee and assessing the characteristics of the bite, including but not limited to periods, places, and circumstances, was administer to the victims.

### Data analysis

The data were organized using Microsoft Office Excel Spreadsheets, and analyzed using the statistical software IBM SPSS Statistics version 20.0 (SPSS Inc., Chicago, IL, USA). The threshold for significance was set to 5% for all the analyses. Categorical variables (sex, occupation, site, place and time of bite) were expressed using percentages, and continuous variables (age) were expressed in term of median (interquartile range, IQR). IMB SPSS statistics “Crosstabs” procedure was used to compute the reporting and non-reporting rates of snakebites that were compared between relevant covariates (gender, age group, occupation, as well as site, place and time of bite) using the Chi-square test.

## Results

### Sociodemographic characteristics of snakebite victims

**Fig. 1:**
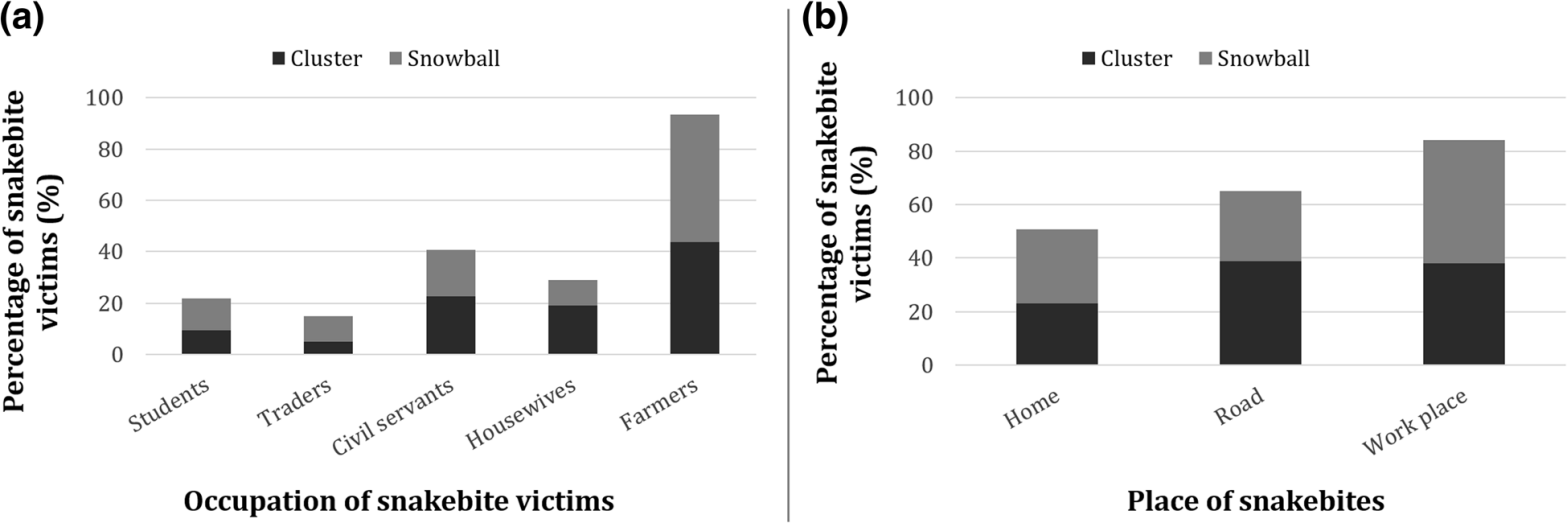
Distribution of snakebite victims according to their socio-occupational categories (**a**) and place where they were bitten (**b**)

**Fig. 2:**
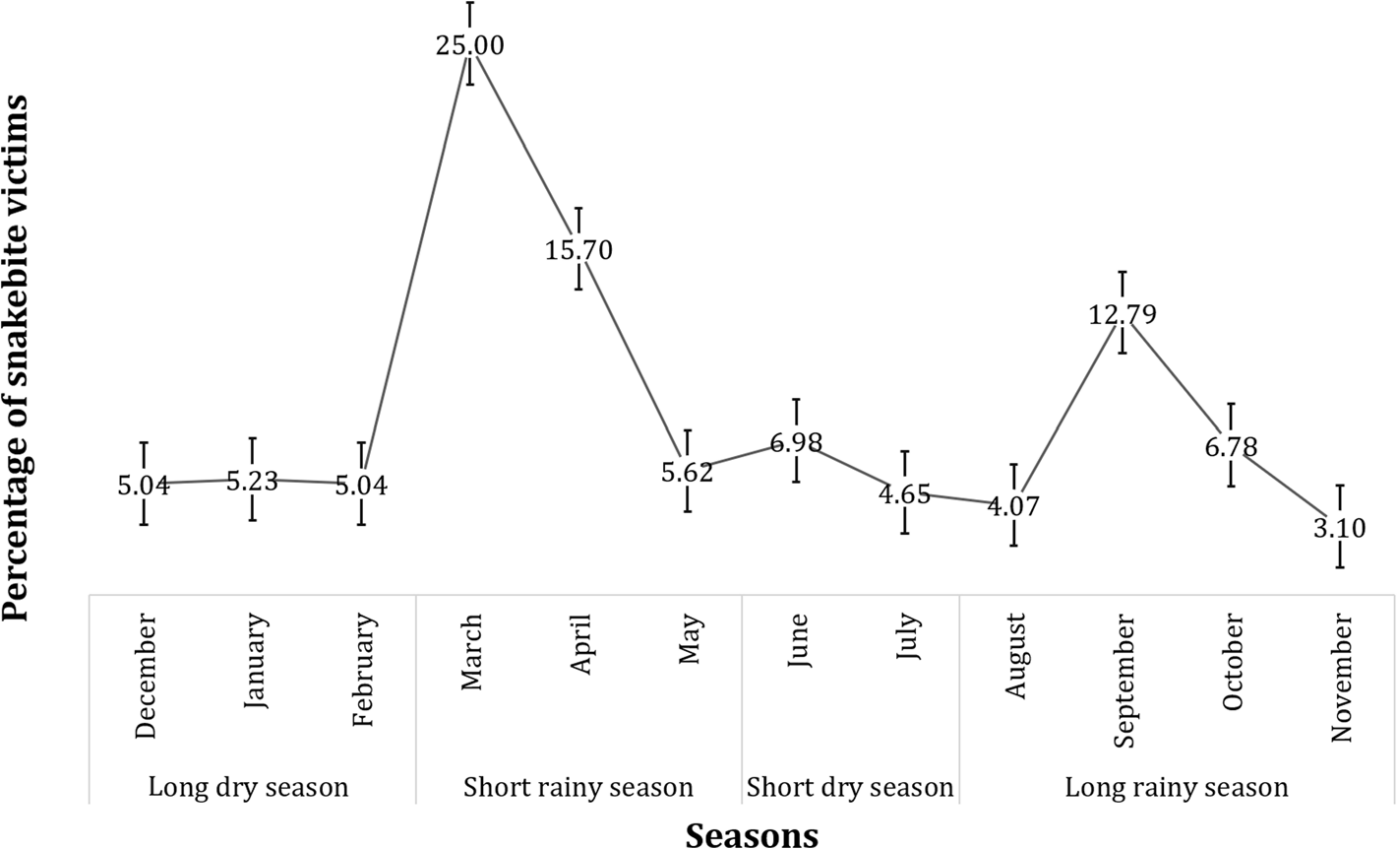
Monthly and seasonal distribution of snakebite cases in the Centre Region

A total of 516 snakebite victims were identified during the study period, with a 2.5% lethality rate. Of note, all victims were attacked by the snake just once. The sex ratio was significantly female biased (61.6%) (Chi square = 55.81; df = 1; *p*-value < 0.0001). The biting rate was significantly higher among individuals aged 31 to 40 years old (40.1%) (Chi square = 72.85; df = 3; *p*-value < 0.0001), the median age of the victims being 32 (IQR: 22–40) years old (Table 1). Regarding the enrollee occupations and the biting place, farmers (42.2%) were significantly more bitten than their counterparts (Chi square = 346.79; df = 6; *p*-value < 0.0001), and most of the enrollees were bitten at their workplace (43.0%) (Chi square = 24.08; df = 2; *p*-value < 0.0001) (Fig. 1). The lower limbs were the most bitten site of the body (64.5%) (Chi square = 565.83; df = 3; *p*-value < 0.0001), and 85.0% of these lower limb bites occur along the road. Most bites occur in the afternoon (51.6%) (Chi square = 86.57; df = 2; *p*-value < 0.0001), and mostly during rainy seasons (73.0%) (Fig. 2).

**Table 1:** Distribution of snakebite victims according to sampling approach, gender and age group

Sampling Approach	Cluster		Snowball		Overall		Total
Age group	Gender		Gender		Gender		
	Males	Females	Males	Females	Males	Females	
< 20	14 (42.4)	19 (57.6)	23 (56.1)	18 (43.9)	37 (50.0)	37 (50.0)	74 (14.3)
21–30	26 (51.0)	25 (49.0)	23 (36.5)	40 (63.5)	49 (43.0)	65 (57.0)	114 (22.1)
31–40	30 (43.5)	39 (56.5)	39 (28.3)	99 (71.7)	69 (33.3)	138 (66.7)	207 (40.1)
> 40	15 (30.0)	35 (70.0)	28 (39.4)	43 (60.6)	43 (35.5)	78 (64.5)	121 (23.4)
Total	85 (41.9)	118 (58.1)	113 (36.1)	200 (63.9)	198 (38.4)	318 (61.6)	516

### Incidence of snakebites

In addition to the cases surveyed in health facilities, 60,7% of the snakebite victims were enumerated and surveyed within households in the targeted health areas. The annual incidence was 36,6 snakebites per 100,000 inhabitants in the study area. The geographical distribution was globally heterogeneous. Indeed, the health areas of Ngallan, Yoko and Nkilzok inside the district of Mbalmayo, Yoko and Mfou were characterized by high incidence rates of 250, 290 and 308 cases per 100,000 inhabitants, respectively. Based on data from the routine collection system, some health districts were characterized by zero annual incidence rates (Ngo-Mapubi and Nkolbisson). However, the study identified bite cases in all health districts of the Centre Region during the same period, and the lowest incidence rate observed during the survey was 2 cases per 100,000 inhabitants of the Nkolndongo health district (Table 2).

**Table 2:** Number of snakebite victims observed and reported, and percentage difference by health district

Health District	Population^a^	N Reported	N Observed	% Diff^b^
Akonolinga	33,712	7	13	46.2
Awae	14,115	3	13	76.9
Ayos	21,507	6	6	0.0
Bafia	26,113	11	28	60.7
Biyem Assi	59,120	7	16	56 .3
Cite Verte	157,687	12	41	70.7
Djoungolo	75,540	14	54	74.1
Ebebda	15,664	3	8	62.5
Efoulan	201,654	5	12	58.3
Elig Mfomo	9404	3	12	75.0
Eseka	22,020	4	6	33.3
Esse	9552	3	17	82.4
Evodoula	12,126	2	10	80.0
Mbalmayo	20,673	6	25	76.0
Mbandjock	35,238	2	5	60.0
Mbankomo	23,035	2	20	90.0
Mfou	37,070	4	14	71.4
Monatele	22,490	3	17	82.4
Nanga Eboko	35,662	6	11	45.5
Ngog Mapubi	12,754	0	14	100.0
Ngoumou	21,640	5	8	37.5
Nkolbisson	43,839	0	10	100.0
Nkolndongo	266,952	2	5	60.0
Ndikinimeki	33,601	10	11	9.1
Ntui	38,781	12	59	79.7
Obala	60,700	8	10	20.0
Okola	12,242	5	13	61.5
Saa	34,920	7	18	61.1
Soa	28,186	8	16	50.0
Yoko	23,311	6	24	75.0
Total	1,409,348	166	516	67.8

^a^: Population of the selected health areas; ^b^: percentage difference or non-reporting rate; *N* number

### Snakebite reporting rate

Overall, 39.3% of the victims were identified in the hospital, and 60.7% were found in communities thanks to the snowball technique. A total of 100 health facilities were visited during the study, among which 74 received at least one case a snakebite victim during the study period. The snakebite victims were mostly bitten in the afternoon (51.7%), and 37.3% of snakes identified by the victim was a forest cobra. Snakebites were predominantly recorded in the health districts of Ntui and Djoungolo (11.3 and 10.5%, respectively) (Chi square = 289.58; df = 29; *p*-value < 0.0001) (Table 2).The percentage difference between the numbers of reported and observed snakebite victims revealed an overall 67.8% of snakebite cases not reported by the health system, the non-reporting rate varying from one health district to another (Table 2).

Regarding the different covariates investigated in this study, the non-reporting rate of snakebite cases was similar between males and females (Chi-square: 1.194; df: 1; *p*-value: 0.275), amongst age groups (Chi-square: 7.039; df: 3; *p*-value: 0.071) and time at which patients were bitten (Chi-square: 4.658; df: 2; *p*-value: 0.097) (Table 3). However, the non-reporting rate was significantly different among (i) occupational categories (Chi-square: 15.107; df: 4; *p*-value: 0.004), traders, students and farmers/breeders exhibiting highest non-reporting rates compared to civil servants and housewives; (ii) religions (Chi-square: 33.782; df: 3; *p*-value < 0.0001), Muslims exhibiting the lowest non-reporting rate; (iii) places where snakebite occurred (Chi-square: 9.294; df: 2; *p*-value: 0.010), people bitten at their workplace or at home reporting the less snakebites; and (iv) site of snakebite (Chi-square: 24.670; df: 3; *p*-value < 0.0001), individuals bitten on the head exhibiting the lowest snakebite non-reporting rates (Table 3).

**Table 3:** Non-reporting rate of snakebite cases according to gender, occupation, age group, religion as well as place, time and site of snakebite

Variable	No. snakebite cases	No. non reported cases (%)	Chi-square (*p*-value)
Gender			
Male	198	126 (63.6)	1.194 (0.275)
Female	318	187 (58.8)	1.194 (0.275)
Occupation			
Housewives	70	31 (44.3)	15.107 (0.004)^b^
Students	58	39 (67.2)	
Traders	41	31 (75.6)	
Civil servants	102	56 (54.9)	
Farmer/Breeders	245	156 (63.7)	
Age groups			
< 21 years old	74	51 (68.9)	7.039 (0.071)
21–40 years old	114	77 (67.5)	
41–50 years old	207	115 (55.6)	
> 50 years old	121	70 (57.9)	
Religion			
Catholicism	376	252 (67.0)	33.782 (< 0.0001)^b^
Protestantism	80	37 (46.3)	
Islamism	21	4 (19.0)	
Other^a^	26	10 (38.5)	
Place snakebite occurred			
Home	133	86 (64.7)	9.294 (0.010)^b^
Road	161	82 (50.9)	
Workplace	222	145 (65.3)	
Time snakebite			
Morning	97	59 (60.8)	4.658 (0.097)
Afternoon	266	172 (64.7)	
Night	152	82 (53.9)	
Site snakebite			
Upper member	169	122 (72.2)	24.670 (< 0.0001)^b^
Lower member	333	183 (55.0)	
Body	9	8 (88.9)	
Head	5	0 (0.0)	

*No.* number of; ^a^: animism and non-believers; ^b^: significant difference

## Discussion

Because of its high burden, snakebite is now among the WHO Neglected Tropical Diseases (NTDs) needing an intervention. This requires an up to date mapping of this neglected condition to prompt better management. In Cameroon where snakebite incidence is among the greatest in Africa, snakebites have been registered among Potential Epidemic Diseases (PED), and weekly reports are elaborated at health districts level for up to date data. The objective of this study was to assess the real snakebites reporting rate in the Centre Region of Cameroon. To do this, a retro-prospective survey was carried out following two sampling techniques, cluster and snowball approaches.

This study reveals that snakebites were more frequent among females, though the sex ratio was highly male biased in others studies [9]. This could be explained by the fact that in this study, the main exposure/risk factor to snakebites, agriculture, is mostly practiced by women. An important increase in the number of snakebite cases was recorded during the day, and especially at the beginning of the rainy season. A similar result has already been observed in Senegal by Chippaux and Diallo where 60% of the cases occurred during the day and 55% during the rainy season [9]. In fact, snakes appear to be disturbed in their biotopes at the beginning of the rainy season, either by the nature of time due to precipitation or by human being involved in intense agricultural activities.

A heterogeneous distribution of snakebite cases was observed by health district and might be explained by the diversity of the environmental facets of this Region which offers to the herpetofauna suitable biotopes for development. It would also be due to the types of activities carried out by the inhabitants. Indeed, the practice of agriculture has been identified as the main risk factor for snakebites in several epidemiological studies conducted in the African context [3, 10]. In this study, the Ntui health district where the highest morbidity rate was observed is one of the main agricultural area in the Centre Region, and it was found that farmers are the most bitten and are most often bitten during their activities. Also, the high proportion of victims among active adults (66.0%) and the relatively high number of accidents during agricultural activities indicate the existence of a rich snake fauna in this study environment. In the studies conducted in sites characterized by a depletion of snakes fauna like the region of Niakhar in Senegal, a lower snakebite rate is observed [9, 11].

The incidence of bites in the study area (36,6 snakebites per 100,000 inhabitants), is nearly five times lower than the 250 per 100,000 usually observed in rural areas of sub-Saharan Africa [11]. This can be explained by the fact that our study site was a semi-rural/semi-urban environment plagued by the rural exodus. However, three rural health areas have similar incidence rates with those commonly found in rural areas of sub-Saharan Africa. The reported morbidity (49 envenomations per 100 victims) is substantially higher than those in rural and Sahelian areas of Senegal [9]. The high morbidity of snakebites in the Centre Region (Cameroon) might be explained by the fact that the study site is located in the South-Cameroon plateau grouping the largest diversity of venomous snakes of the country [6].

This survey shows that the frequentation of modern health facilities by snake bitten victims is limited. This phenomenon is not specific to the Centre Region of Cameroon, but is observed in many other countries, particularly in Guinea [12], Senegal [9], and Benin [13]. This study highlights the limitations of this very widely used source of information in Cameroon where modern medicine is not the only therapeutic remedy used in the event of a snakebite, and thus revealed a high non-reporting rate. The collection of epidemiological information on envenomations should not be therefore restricted to the registration and notification of cases attending health facilities. This high rate of non-reporting of snakebite cases might be explained by the fact that the actors (including health professionals) involved in the data collection are not aware of the existence of the new PED data collection form set up in 2015, with a specific item on snakebite cases, and/or do not master the functioning of the updated national system of surveillance of PED. Also, not all the stakeholders involved in snakebite management are associated in the process of snakebite reporting, particularly traditional healers.

The non-reporting rate of snakebites was the highest among (i) farmers/breeders, students and traders, (ii) Catholics and Protestants, and (iii) among individuals bitten at home or at their workplace. Indeed, it has been demonstrated that in rural areas, snakebites usually occur in farms, far from health facilities. Thus, distance from place where snakebites occur and health facility might constitute a limiting factor to reporting, an envenomed victim needing to walk (or to be carried) for many miles to reach a primary health facility [14]. Also, these high non-reporting rates may be due to the orientation of the victims towards the traditional healers who are more accessible, especially in rural areas where health facilities are usually far [15]. Finally, the site of snakebite was found to influence decision to report or not, likely because of the ability to take action (tourniquet for example); indeed, all individuals who were bitten on the head reported, contrarily to those bitten on the members, either lower and upper.

## Conclusion

Snakebite is endemic in the Centre Region of Cameroon, the high-risk population being females aged 30 years and over. Although agricultural activities are the main factors of human-snake encounter and farmers remain the most bitten population as in other rural areas of Africa, a reduced number of snakebites occurred in other socio-professional categories. The victims were oriented in priority to traditional medicine, suggesting that the modern system of treatment is limited to describe all the snakebites cases. Considering the high underreporting rate observed in the framework of this study, the various sources of information could be complementarity to update and refine existing data.

## Data Availability

The datasets used and/or analyzed during the current study are available from the corresponding author on reasonable request.

## Abbreviations

AVS: Anti-Venom Serum
CFR: Case fatal rate
IST: International Society of Toxinology
MoPH: Ministry of Public Health
NSAID: Non-steroidal anti-inflammatory drugs
NTD: Neglected Tropical Diseases
PED: Potential Epidemic Diseases
WHO: World Health Organization

## References

[1] WHO: Global snakebite burden, report by the director-general. In: https://apps.who.int/iris/handle/10665/276406 2017 edn. Geneva: WHO; 2017.

[2] WHO: Snakebite envenoming. WHO Fact sheet 2017.

[3] Chippaux JP (2011). Estimate of the burden of snakebites in sub-Saharan Africa: a meta-analytic approach. Toxicon.

[4] Mion G, Olive F, Giraud D, Lambert E, Descraques C, Garrabé E, Goyffon M. Surveillance clinique et biologiquedes patients envenimés. Bull Soc Pathol Exot. 2002;95(3):139–143. 12404854

[5] Chippaux JP, Massougbodji A: morsures de serpent en Afrique un problème de santé publique. In: https://www.ird.fr/la-mediatheque/fiches-d-actualite-scientifique/374-morsures-de-serpents-un-probleme-de-sante-publique-en-afrique IRD; 2011: 2–1.

[6] Gonwouo NL, LeBreton M, Chirio L, Ngassam P, Ngoa LE (2005). G. D: Répartition biogéographique des serpents venimeux au Cameroun. Bull Soc Pathol Exot.

[7] Kasturiratne A, Wickremasinghe AR, de Silva N, Gunawardena NK, Pathmeswaran A, Premaratna R (2008). The global burden of snakebite: a literature analysis and modelling based on regional estimates of envenoming and deaths. PLoS Med.

[8] MINSANTE: Stratégie sectorielle de santé 2016–2027 http://www.nationalplanningcycles.org/sites/default/files/planning_cycle_repository/cameroon/cameroon_-_sss_validee_par_le_ccss_5_janvier.pdf; 2016.

[9] Chippaux JP, Diallo (2002). Évaluation de l’incidence des morsures de serpent en zone de sahel sénégalais, l’exemple de Niakhar. Bull Soc Pathol Exot.

[10] Gutierrez JM, Warrell DA, Williams DJ, Jensen S, Brown N (2013). The need for full integration of snakebite envenoming within a global strategy to combat the neglected tropical diseases: the way forward. PLoS Negl Trop Dis.

[11] Chippaux JP (1999). L’envenimation ophidienne en Afrique : épidémiologie, clinique et traitement. Ann IP/actualités.

[12] Baldé MC, Camara AMB, Bah H, Barry AO, Camara SK (2005). Incidence des morsures de serpent: enquête communautaire dans la collectivité rurale de développement (CRD) de Frilguiagbé (République de Guinée). Bull Soc Pathol Exot.

[13] Chippaux JP (2002). Epidémiologie des morsures de serpent au Bénin. Bull Soc Pathol Exot.

[14] Eliza S, Mohammad AM, Mya MK, Dale H, Khin TT, Nyein NC, Robert C, David B, Sam A, Julian W, David W, Chen AP. Why snakebite patients in Myanmar seek traditional healers despite availability of biomedical care at hospitals? Community perspectives on reasons. PLoS Negl Trop Dis. 2018;12(2). 10.1371/journal.pntd.0006299 PMC5847227 29489824

[15] White P. The concept of diseases and health care in African traditional religion in Ghana. HTS Theolog Stud. 2015;71(3).

